# Strengthening the referral Chain and providing one window diabetes eye care facility to people with Type-2 Diabetes: A six-year follow-up study from Pakistan

**DOI:** 10.12669/pjms.37.7.3946

**Published:** 2021

**Authors:** Shahid Ahsan, Muhammad Saleh Memon, Muhammad Faisal Fahim, Tauseef Mahmood, Sikander Ali Sheikh

**Affiliations:** 1 Dr. Shahid Ahsan, MPhil (Bio), MPhil (NCD), PhD fellow (KU) Department of Biochemistry, Jinnah Medical & Dental College, Karachi, Pakistan; 2 Dr. Muhammad Saleh Memon, FRCS(Eden) Department of Research, Al-Ibrahim Eye Hospital, ISRA Postgraduate Institute of Ophthalmology, Karachi, Pakistan; 3 Mr. Muhammad Faisal Fahim, M.Sc. (Statistics) Department of Physical Therapy, Bahria University Medical & Dental College, Karachi, Pakistan; 4 Mr. Tauseef Mahmood, M.Sc. (Statistics) Department of Research, Al-Ibrahim Eye Hospital, ISRA Postgraduate Institute of Ophthalmology, Karachi, Pakistan; 5 Mr. Sikander Ali Shaikh, M.A (Sociology) Department of Outreach Programs, Al-Ibrahim Eye Hospital, ISRA Postgraduate Institute of Ophthalmology, Karachi, Pakistan

**Keywords:** Comprehensive eye care, Diabetic retinopathy, Referral source, Type-2 Diabetes Mellitus

## Abstract

**Objective:**

To report the results of implementation of two-pronged system for strengthening of referral and receiving end of referral chain for people with Type-2 diabetes mellitus (T2DM) at a tertiary eye care hospital in Karachi.

**Methods:**

This observational, cross sectional study was conducted from the data collected in “Strengthening Pakistan’s response to Diabetic Retinopathy” project. Peripheral referral was improved through community awareness camps (n=48), refreshing knowledge of primary healthcare professionals (PHCP) and conducting retinopathy screening camps (n=85) in the community. T2DM patients with best corrected visual acuity (BCVA) <6/18 or had retinopathy sign on screening were referred to tertiary center. The receiving end of the referral was improved by establishing “one window facility” at tertiary eye care center. Facility consisted of eight stations starting from registration, visual assessment, fundus photographs, consultation with ophthalmologist, anthropometry measurement, consultation with diabetologist to finally meeting with diabetes educator. At every station, patient’s information was directly entered in HIMS software.

**Results:**

A total of 50,595 patients attended tertiary center over six years. Among all 34685 (68.5%) were new registration and 15910 (31.4%) were follow ups. During first year (2014-15) out of total registered individuals with DM, newly registered were 4414 (89.5%) and 518 (10.5%) were follow-ups. In the final year (2019-20) new cases registered reached to 62% (n= 7916) with 38% (n=4852) follow-ups. Patients referred by PHCP increased from 6.5% in 2014-15 to 43.7% in 2019-20. An increased uptake of all treatment modalities for retinopathy like laser (increased by 16.76%), intra-vitreal injections (by 14.72%) and vitrectomy (by 51.47%) were also observed.

**Conclusion:**

Implementation of two prong system resulted in improved service uptake, better referral system, enhanced follow-ups and increased intervention uptake.

## INTRODUCTION

Chronic complications of Type-2 DM have insidious onset.^1^ Once developed; it remains unnoticed till the disease is well advanced to produce symptoms. International organizations therefore recommend annual screening of these complications for tertiary prevention of diabetic complications. Early detection and prompt treatment halts or even regresses the progression of this complications.^2^ Despite recommendations and documented benefits, uptake of screening facilities in developing countries has yet not reached the optimal levels.^3^ Low uptake of health care services is a general phenomenon in developing countries.^4^ According to WHO only a quarter of people in need of eye care, use eye care services.^5^

Reasons for low uptake of health facilities vary from country to country and region to region. In low and middle-income countries like Pakistan, people in general have poor education and low health literacy. There is generally a lack of awareness about importance of screening. The incongruous health beliefs and misconceptions of the community also affect their attitude towards uptake of services. This situation further lowers the acceptance of appropriate treatment after screening and causes loss to follow up. These barriers along with low or inappropriate referral, un-even distribution of skilled personnel, overburdened specialists in tertiary centers and inadequate resources in higher health care facilities are the challenges for the provision of appropriate post screening treatment.^3^

With escalating prevalence of diabetes worldwide,^6^ screening of chronic diabetic complications, particularly retinopathy screening has become a matter of concern for public health workers since beginning of the twentieth century.^3^ In the developed countries, diabetic retinopathy (DR) has emerged as one of the major causes of vision impairment.^7^ In Pakistan, uncorrected refractive errors, cataract, and glaucoma,^8^ once surpassed retinopathy (DR) as common conditions of vision impairment and blindness. Now it is widely realized that in the last decade the frequency of patients being diagnosed with diabetes and subsequently with DR has increased incrementally in the country.^9^ Recent survey has shown that prevalence of diabetes in Pakistan is 26.3%, of which 19.2% are those with known DM.^9^ Among patients with DM, studies have reported that 20-26% have retinopathy and 8% have sight threatening DR.^10^,^11^

Considering the increased prevalence and silent nature of this vision threatening complication of diabetes, the traditional approach where optimum glycemic, lipids and blood pressure control is focused and managed by one specialist and the coexisting complications are either overlooked or managed by different specialists at different places without any coordination, is not desirable.

For proper management of individuals with DM, there is a need for multidisciplinary approach. To achieve this objective, two prong system including referring end and the receiving end of the referral chain was developed. Improvement of awareness in the community and education of the service providers about management of DM resulted in strengthening of referring end of the chain. Establishment of “One window facility” at Al-Ibrahim Eye Hospital (AIEH) reinforced the receiving end of the chain. The present study highlights the results of this system.

## METHODS

A project “strengthening Pakistan’s response to Diabetic Retinopathy” was initiated in district Malir, Karachi from April 2014 till March 2020. This observational, cross sectional study was conducted from the data collected during the project, after ethical approval at AIEH. During the project two-pronged system was established in order to strengthen the peripheral (referral end) as well as receiving end of referral the referral system.

Peripheral referral was improved through awareness in the community, enhancing knowledge of service providers and developing linkages with them. The receiving end of the referral was improved by establishing “one window facility” at tertiary eye care center.

### One-day training for Medical Officers, General Practitioners and LHW’s

To start with one-day structured training was arranged for Medical Officers, General Practitioners, Lady Health Workers and Lady Health supervisors at nearby health facilities in district Malir, Karachi. The main objective of the training was educating health professionals for DM and diabetes related complications with especial reference to Diabetic retinopathy and early referrals of known diabetics for retinal screening to tertiary eye care hospital.

A total of 40 Medical Officers (MO), 55 General Practitioners (GP), 622 Lady Health Workers (LHW) and 24 Lady Health Supervisors (LHS) attended the sessions. At middle of the project, refresher training session was held.

### Awareness Raising Sessions

A total of 48 awareness raising sessions were conducted in different locations in district Malir, Karachi by trained health professionals and project team in their catchment areas. Awareness sessions were conducted separately for general community (Group-A) and for people with known DM (Group-B).

### Group-A

In the sessions for general community, awareness was given about DM, its signs and symptoms, risk group, prevention of DM and key messages on life style modifications like, healthy eating, avoidance of junk foods and importance of physical activity for prevention of DM.

### Group-B

In awareness sessions of this group, information was given regarding chronic complications of DM, especially retinopathy, its risk factors like hypertension, blood lipids and blood sugar level and importance of periodic screening of these factors. Importance of Self-Monitoring Blood glucose (SMBG), signs and symptoms of hypo- and hyper-glycaemia, optimal targets for glycemic control, adequate diet, physical activity, and importance of early screening of diabetic complications was discussed.

### DR Screening Camps in the Community

To enhance the referral of the patients with DM having diabetic retinopathy, periodic camps were held in various primary health care facilities in district Malir. A total of 85 screening camps were carried out in the community during the project. Direct ophthalmoscopy by optometrist was carried out in all known diabetics attending the camps after dilating the pupil with 1% Tropicamide. All the individuals having best corrected visual activity of less than 6/18, invisible fundus and any sign of Diabetic retinopathy were referred to the tertiary centre for confirmation, grading and treatment.

### Establishment of One Window comprehensive eye care facility for people with DM

AIEH is located in district Malir, a tertiary eye care facility acted as receiving end of the referral chain. One window facility was established in 2014 to cater for the patients with DM. Facility consisted of eight stations starting from registration, visual assessment, fundus photographs, consultation with ophthalmologist, anthropometry measurement, consultation with diabetologist to finally meeting with diabetes educator. At every station, patient’s information was directly entered in HIMS software. All patients visiting on their own or referred by various Primary Health care providers were facilitated at an exclusive registration desk. Data was preserved electronically with a unique registration number and source of referral. Demographic data, visual assessment, digital Fundus images on Non Mydriatic Fundus Camera, slit lamp bio-microscopy with +90D fundus examination was performed. DR (this abbreviation must be used where the term diabetic retinopathy was used the first time. These are basics of writing) diagnosis with grading was made and managed accordingly. DM counselling was done by Certified Diabetes Educator. Individuals with ocular pathologies were referred to respective specialists for appropriate management under one roof. Random blood sugar (RBS) was checked with glucometer, Blood pressure, height and weight of all patients were recorded. Management of DM was done by General Physician. In 2016, diabetologist was introduced to manage DM, screening and basic treatments of diabetic neuropathy (DN), diabetic nephropathy (DN) and diabetic foot (DF). Biochemical tests like RBS, HBA1c, Lipid Profile, Serum Creatinine and Urine DR were made available in laboratory. Patient’s status was communicated to the referring source. Follow-up calls were made one day prior to the appointment date.

### Statistical Analysis

Data was retrieved from Hospital Information Management System (HIMS) and exported to SPSS version 23.0. Frequencies and percentages were reported in trend chart (year wise) and in graphs for Service acceptance, Referral Source, follow up and treatment uptake for retinopathy.

## RESULTS

### Service acceptance

A total of 50,595 patients attended during the study period of six years. New registration was 34685 (68.5%) with 15910 (31.4%) follow ups. Male to female ratio of newly registered individuals was 1.1: 1, with 18245 (52.60%) male and 16440 (47.40%) female. Most of the participants, 18363 (52.96%) were in the age group of 46-60 years.

**Fig.1:**
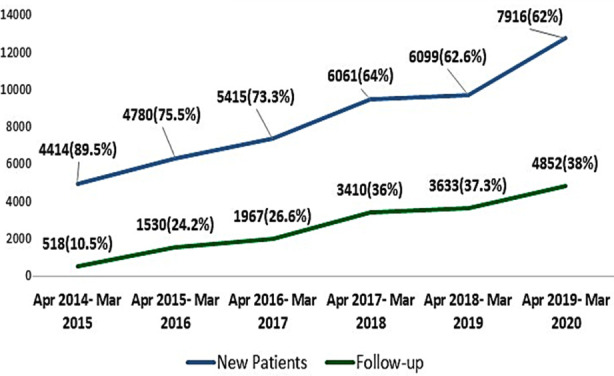
Service acceptance – Frequency of New Registration and Follow ups.

During first year of the project (2014-15) newly registered individuals with DM were 4414 (89.5%) with 518 (10.5%) follow-ups. In the final year (2019-20) new registration was 7916 (62%) with 4852(38%) follow-ups (Fig.1).

### Referral pattern

During first year of the project, 3867 (87.6%) individuals out of total of 4414 attended on their own, (whereas 291 (6.5%) were referred by primary health professionals- LHW’s 35 (0.79%), MO’s 192 (4.3%) and GP’s 65 (1.4%)), while another 256 (5.7%) were referred through camp screening.

**Fig.2:**
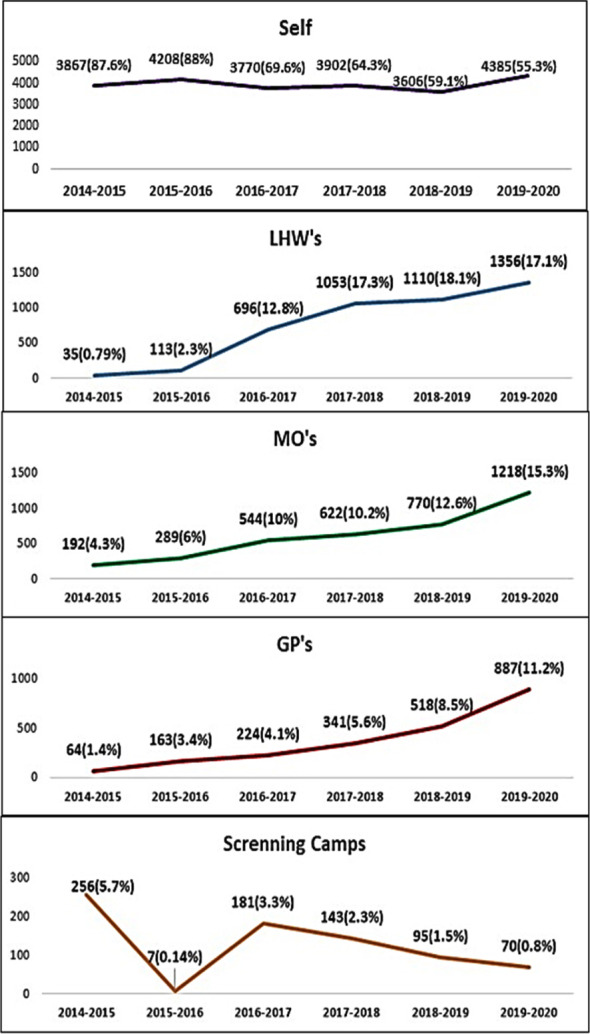
Frequency of patients referred to tertiary care center by different sources.

In the last year (2019-20), out of 7916 newly registered patients, 4385 (55.3%) attended on their own, and 3461 (43.7%) patients were referred by different primary health professionals- (LHW’s 1356 (17.1%), MO’s 1218 (15.3%) and GP’s 887 (11.2%)), while a further 70 (0.8%) patients were referred through camp screening (Fig.2).

### Follow ups

**Fig.3:**
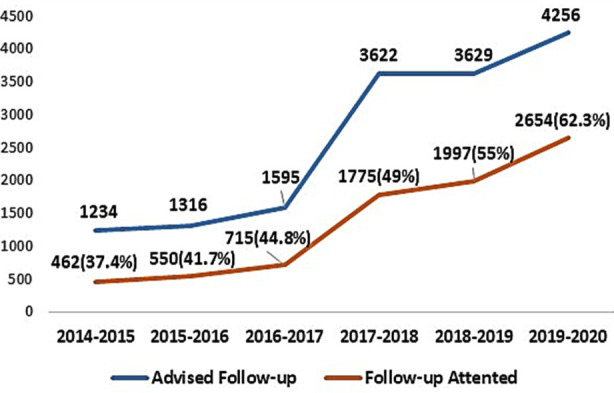
Follow-up Advised and Accepted.

Follow-up is defined as the attendance of an individual within one month of the advised date. During the study period number of follow-up patients increased from 37.4% in 2014-15 to 62.3% in 2019-2020, reflecting a rise of 24.9% over the period of six years (Fig.3).

### Treatment Acceptance

**Fig.4:**
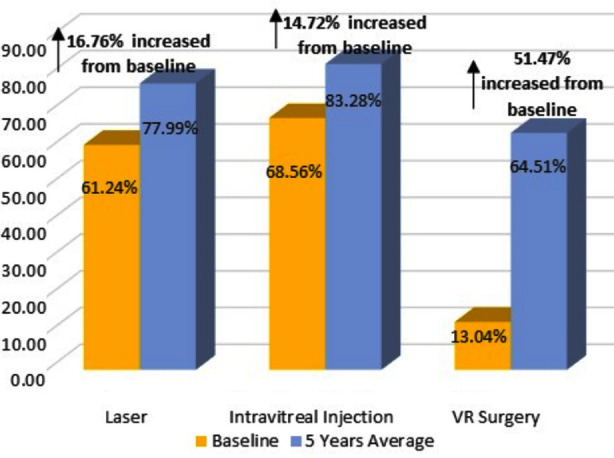
Treatment Uptake for Retinopathy.

At baseline, laser acceptance was 61.24%, intravitreal injection acceptance was 68.56% and VR surgery acceptance was 13.04%. At 5-year average upto final year, acceptance was found to be 77.98% in laser, 83.28% in intravitreal injection and 64.51% in VR surgery. An increased uptake of all treatment modalities for retinopathy like laser (increased by 16.76%), intra-vitreal injections (by 14.72%) and vitrectomy (by 51.47%) were also observed (Fig.4).

## DISCUSSION

Implementation of two prong system over the period of six years, showed gradual rise in the registration of new and follow-up patients, increased number of successful referrals from primary to tertiary health center and increased uptake of advised interventions.

Referral failure is one of the major obstacles in the delivery of health care services^12^ A sound referral system from primary to secondary / tertiary care health facility is imperative to cater the rapidly increasing number of diabetics and escalating burden on health economy.^13^ The core concept of this design is to encourage the patients to first seek advice at the primary level, and then approach a higher level of care according to the need. This minimizes the costs on the patients and burden on health care provider and higher center resources. However, in most of the cases, patients bypass the primary facilities and self-refer themselves to the higher center. Vice versa, some of the patients who required specialized care failed to get referring advice or did not show compliance to the advice. Thus, a patient in need of expert opinion skips getting the specialized treatment and advice. Even the developed countries had struggled to reduce the referral failure rate that varies widely across the world.^14^,^15^ British national health services had reported referral failure rate as 12%^14^ whereas in Western Uganda it was reported to be 31%.^15^ Various studies originated from Pakistan have identified poor knowledge, limited awareness and indifferent attitude towards the disease as major causes of referral failure particularly for people living in rural and remote areas.^16^,^17^ These obstacles along with inadequate services, non-coordination amongst the service providers, unaffordability of the patients and absence of counselling at tertiary service level result in lack of treatment acceptance and drop in follow ups.^18^,^19^

Two prong system was initiated in April 2014 to address these challenges. One prong of the system was dedicated to strengthen the referring end through “Diabetes education program (DEP)”. Primary care provider like LHWs, medical officers of the public health sectors and family physicians in the private sector were mobilized through awareness campaign. Knowledge of primary care providers regarding diabetes and its chronic complications was reinforced by the Diabetes Educator. The primary health care professionals in turn created awareness amongst general community. This strategy improved referrals from primary health care facility to the tertiary level. The results of this approach show gradual increase in registration of new individuals with diabetes at tertiary eye care center. Similar change in frequency of referrals by various referring source (lady health workers, medical officers, general practitioners) was also observed during the study period.

Second prong was directed to strengthen the receiving end of the referral system. One window facility was established to provide comprehensive care to the individual with DM. Diabetes focused registration counter ensured zero loss of the individual with DM attending the tertiary eye care center. It was thought that this will facilitate the referred individuals. Reminder call via help line telephone service a day before the appointment together with counselling resulted in nearly nine- fold rise in the number of follow up patients during the period of six years (Fig.1). Counselling by the diabetes educator improved the treatment acceptance.

## CONCLUSION

Implementation of two prong system resulted in improved service uptake, better referral system, enhanced follow-up and increased intervention uptake.

### Authors Contribution

**MSM** conceived, designed and manuscript writing & final approval. He is also responsible and accountable for the study.

**SA** did design of study, manuscript writing & critical review.

**MFF** did statistical analysis, Results writing & critical review of manuscript.

**TM** did data collection, summarizing of data & editing of manuscript.

**SAS** did data collection and methodology writing.
